# Magnetic resonance image findings in pug dogs with thoracolumbar myelopathy and concurrent caudal articular process dysplasia

**DOI:** 10.1186/s12917-019-1866-0

**Published:** 2019-05-31

**Authors:** Colin J. Driver, Jeremy Rose, Anna Tauro, Ricardo Fernandes, Clare Rusbridge

**Affiliations:** 1 Fitzpatrick Referrals Ltd, Eashing, Godalming, Surrey, GU7 2QQ UK; 2 Faculty of Health & Medical Sciences, University of Surrey School of Veterinary Medicine, Daphne Jackson Road, Guildford, Surrey, GU2 7AL UK; 3 Lumbry Park Veterinary Specialists, Selborne Road, Alton, Hampshire GU34 3HL UK

**Keywords:** MRI, Facet dysplasia, Vertebral malformation, Pug dogs

## Abstract

**Background:**

A retrospective case series study was undertaken to describe the magnetic resonance imaging (MRI) findings in Pug dogs with thoracolumbar myelopathy and concurrent caudal articular process (CAP) dysplasia. Electronic clinical records were searched for Pug dogs who underwent MRI for the investigation of a T3-L3 spinal cord segment disease with subsequent confirmation of CAP dysplasia with computed tomography between January 2013 and June 2017. Clinical parameters age, gender, neuter status, body weight, urinary or faecal incontinence, severity and duration of clinical signs were recorded. MRI abnormalities were described. Univariable non-parametric tests investigated the association between the clinical parameters and evidence of extra- or intra-dural spinal cord compression on MRI.

**Results:**

18 Pug dogs were included. The median age was 106 months with median duration of clinical signs 5 months. All presented with variable severity of spastic paraparesis and ataxia; 50% suffered urinary/faecal incontinence. In all cases, MRI revealed a focal increase in T2-weighted signal intensity within the spinal cord at an intervertebral level where bilateral CAP dysplasia was present; this was bilateral aplasia in all but one case, which had one aplastic and one severely hypoplastic CAP. MRI lesions were associated with spinal cord compression in all but one case; intervertebral disc protrusion resulted in extra-dural compression in 10 (56%) cases; intra-dural compression was associated with a suspected arachnoid diverticulum in 4 (22%) cases and suspected pia-arachnoid fibrosis in 3 cases (17%). There was no association between clinical parameters and a diagnosis of intra-dural vs extra-dural compression. CAP dysplasia occurred at multiple levels in the T10–13 region with bilateral aplasia at T11–12 most often associated with corresponding spinal cord lesions on MRI.

**Conclusions:**

All Pugs dogs in this study were presented for chronic progressive ambulatory paraparesis; incontinence was commonly reported. Although intervertebral disc disease was the most common radiologic diagnosis, intra-dural compression associated with arachnoid diverticulae/fibrosis was also common. Bilateral CAP aplasia was present in all but one Pug dog at the level of MRI detectable spinal cord lesions. A causal relationship between CAP dysplasia and causes of thoracolumbar myelopathy is speculated but is not confirmed by this study.

## Background

The prevalence of neurologic disease in Pug dogs presented to primary care practices in the UK in 2013 was 5.25%, with spinal cord disease representing 1.39% of cases [1]. Pugs appear over-represented for certain spinal disorders, particularly those of developmental and degenerative aetiology. These include congenital [2] and acquired [3–5] spinal arachnoid diverticulae (SAD) and malformations of the thoracic vertebral bodies [4, 6–8]. ‘Constrictive myelopathy’ is a spinal cord disease previously described in Pug dogs, which appears to be associated with caudal articular process (CAP) dysplasia of the post-diaphragmatic thoracic vertebrae [4]. Pug dogs with constrictive myelopathy were diagnosed with intra-dural spinal cord compression associated with fibrous adhesions originating from the dura mater that adhere to the pia; chronic, low-grade vertebral instability associated with CAP dysplasia has been proposed to contribute to arachnoid fibrosis [4]. ‘Lateral narrowing of the spinal cord through T11-L1’ is the only described MRI abnormality in a small number of cases to date [4]. This may be partly related to the difficulty in identifying CAP dysplasia on MRI studies alone. In a recent study utilising CT examinations from neurologically normal Pug dogs, a greater tendency for CAP aplasia (as opposed to hypoplasia) in the post-diaphragmatic region of the thoracic spine in comparison to other similar dog breeds, was identified [9]. This could result in an increased likelihood of CAP dysplasia being clinically relevant in this breed.

A detailed description of MRI and CT findings in Pug dogs with thoracolumbar myelopathy and concurrent CAP dysplasia is lacking. It is also unknown what radiologic diagnoses are more likely to be associated with clinical features such as severity or duration of clinical signs. The purpose of this retrospective case series is to describe spinal MRI findings in Pug dogs presenting with thoracolumbar myelopathy that were additionally examined for the presence and distribution of caudal thoracic CAP dysplasia (hypoplasia and aplasia) with CT. In addition, we investigated the potential association between clinical parameters (age, body weight, sex, chronicity of clinical signs, severity of clinical signs, the presence of incontinence) and whether spinal cord compression is suspected to be extra-dural or intra-dural.

## Methods

Electronic patient records of Pug dogs presenting to Fitzpatrick Referrals Ltd. (Surrey, UK) between January 2013 and June 2017 were reviewed. Inclusion criteria included complete medical records, clinical signs compatible with a T3-L3 myelopathy, and complete MRI and CT studies of the thoracolumbar spine. In this time period, all Pug dogs that presented to our clinic with thoracolumbar myelopathy underwent imaging with both modalities.

Information from medical records retrieved for analysis included patient age, sex, neutering status, duration of clinical signs (months), severity of neurologic deficits using a 15-point functional scoring system previously published elsewhere [10] that was assigned at the time of examination, the presence or absence of urinary and/or faecal incontinence and whether the SOD-1 genetic test (for allelic mutations potentially associated with the development of degenerative myelopathy) was performed and the result thereof.

Patients underwent routine MRI scanning of the thoracolumbar spine using a high-field scanner (1.5 T; Siemens Symphony Tim system, Enlargen Germany) under general anaesthesia in dorsal recumbency. The imaging protocol included sagittal and transverse planes in T1-weighted (TR 550 ms, TE 12 ms, FOV 280 mm, matrix 512 × 448); dorsal plane in Short-tau Inversion Recovery (TR 3400 ms, TE 40 ms, FOV 300 mm, matrix 384 × 270); sagittal and transverse plane in T2-weighted (TR 3650 ms, TE100ms, FOV 280 mm, Matrix 512 × 448) images. To aid in the identification of intra-dural spinal cord compression, the protocol also included three-dimensional constructive interference in steady state (3D-CISS) images that were acquired in a transverse plane (TR 11.5 ms, TE 5.75 ms, FOV 170, Matrix 256 × 256, slice thickness 1 mm). With the aid of the multiplanar reconstruction software integrated to the MRI scanner, 3D-CISS data was further processed in sagittal and dorsal planes. Post-contrast images were not obtained. CT examinations were performed of the entire thoracolumbar spine using a 160-slice scanner (Aquilion Prime Toshiba, Japan) with dogs positioned in dorsal recumbency, using the following exposure parameters: 120 kV, 120-150mAs, 200 mm FOV. The raw data was processed using the bone algorithm, which was subsequently evaluated in three orthogonal planes and three-dimensional volumetric reconstruction.

The MRI and CT scans were assessed using an open-source PACS Workstation DICOM viewer (Osirix Imaging Software, v 3.9.2, Pixmeo, Geneva, Switzerland). MRI abnormalities were recorded according to widely accepted descriptions. These included intramedullary spinal cord lesions (increase in intra-medullary T2-weighted signal intensity at the affected level), intervertebral disc degeneration (loss of normal T2-weighted signal intensity from the nucleus pulposus) [11], intervertebral disc protrusion (smooth bulging of the annulus fibrosus and dorsal longitudinal ligament to result in loss of epidural fat signal dorsal to the disc and extra-dural spinal cord compression) [11], pre-syrinx or syringomyelia (diffuse poorly marginated increase in T2-weighted signal intensity within the dorsal funiculus of the spinal cord cranial to the affected level, or a cavity containing fluid with signal characteristic similar to cerebrospinal fluid, CSF) [12], and whether spinal cord lesions were apparent at one level or multiple levels. SAD formation was suspected to occur where a single expansion (or ‘out-pocketing’) of the dorsal subarachnoid space, containing CSF only, had occurred to smoothly contour the shape of the spinal cord, thus causing intra-dural extra-medullary spinal cord compression [13]. Pia-arachnoid fibrosis was suspected to occur where multiple linear hypo-intense bands appeared to transverse from the arachnoid to the pia across the arachnoid space that grossly distorted the shape of the spinal cord, causing CSF to accumulate in multiple pockets. This was consistent with a previous surgical description [14]. In each case a putative imaging diagnosis was recorded and spinal cord compression (if present) was classified as extra or intra-dural. The CT scans were reviewed to determine if there was unilateral or bilateral CAP dysplasia at the level of concurrent spinal cord lesions on MRI, and if there were other sites of CAP dysplasia that did not correspond to spinal cord lesions on MRI. Dysplastic articular processes were additionally recorded as being hypoplastic or aplastic.

In order to investigate the potential association between clinical characteristics (patient age, body weight, the duration of clinical signs, the presence of faecal or urinary incontinence and the severity of clinical signs) and extra vs intra-dural spinal cord compression, statistical analysis was performed. One case without spinal cord compression was excluded from analysis. Due to the small sample size, statistical analysis used univariable non-parametric tests including Mann-Whiney U and Fisher’s Exact tests for continuous and categorical explanatory variables respectively. All analysis was performed in Stata 14.2 (StataCorp, College Station, Texas, USA).

## Results

Eighteen Pug dogs with thoracolumbar myelopathy met the inclusion criteria in the study period. The ratio of male: male neutered: female: female neutered was 6:4:1:7. The median age at presentation was 108 months (range 60–144), approximately nine years of age. The median duration of clinical signs was 5 months (range 1–30). All Pug dogs presented with ambulatory, spastic paraparesis with proprioceptive ataxia in the pelvic limbs; there was little variation in the severity of the clinical signs although some Pug dogs tended to make mistakes in paw placement more commonly. The median functional score was 10 (range 8–12). Urinary and/or faecal incontinence was common with 9/18 (50%) being affected. Of these 9 dogs, 1 was urinary incontinent, 4 dogs had faecal incontinence and 4 suffered both forms. 8 dogs were tested for the SOD-1 mutation associated with degenerative myelopathy; two were heterozygous and six were homozygous normal.

**Fig. 1:**
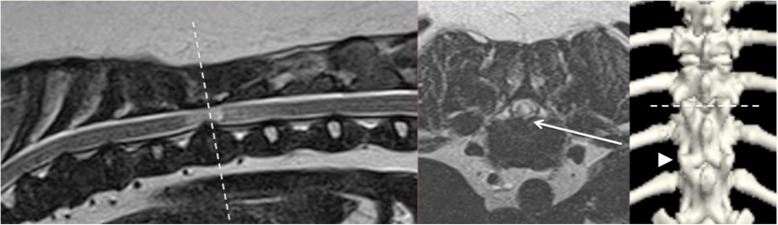
Magnetic resonance imaging of the thoracolumbar spine of case no. 6, displaying extra-dural spinal cord compression associated with T11–12 disc protrusion and concurrent bilateral T11 caudal articular process aplasia. From left to right; T2-weighted mid sagittal of thoracolumbar spine, T2-weighted transverse at the affected level T11–12 (transverse level marked with a dashed line), three-dimensional reconstruction of thoracolumbar junction on CT (T11–12 marked with dashed line; the normal articular processes of T12 are highlighted with an arrow head). There is a marked focal increase in T2-weighted signal within the spinal cord despite only mild ventral extra-dural spinal cord compression (arrowed on transverse image)

**Fig. 2:**
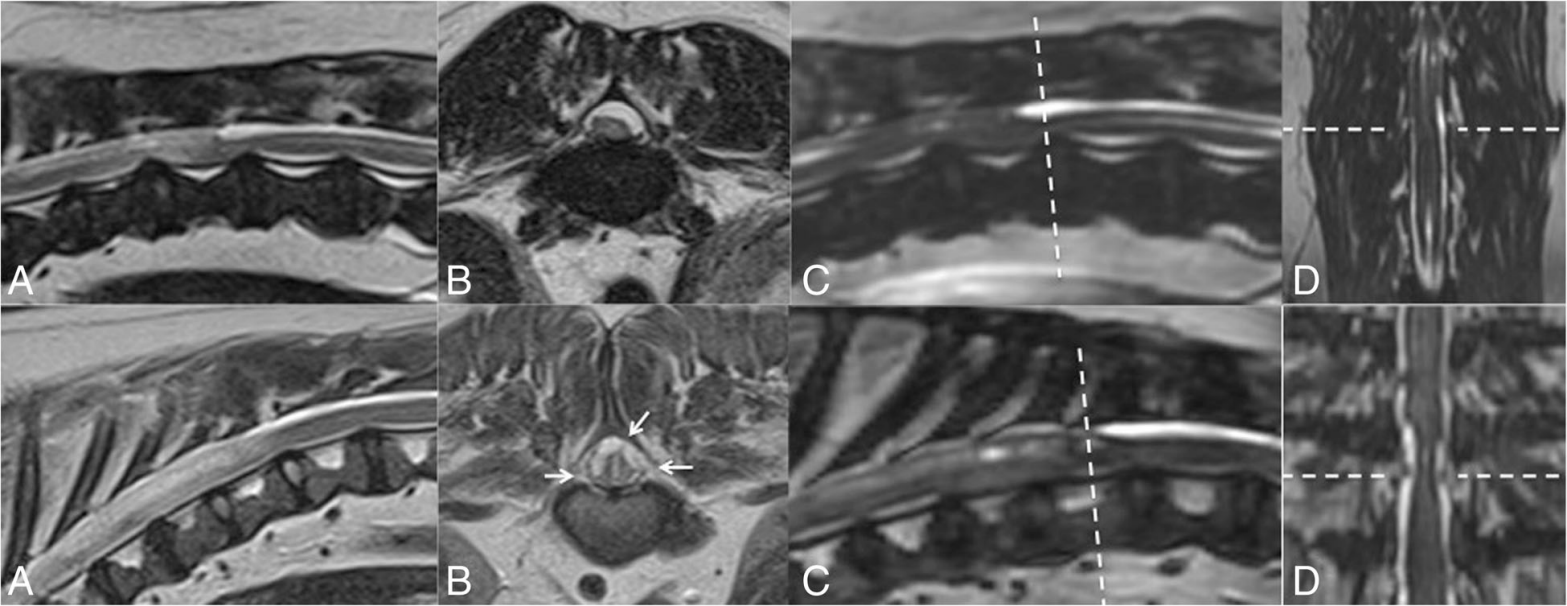
Magnetic resonance imaging of the thoracolumbar spine of case no. 8 (top row) and 14 (bottom row) who display intra-dural spinal cord compression. From left to right; **a** = T2-weighted sagittal MRI, **b** = T2-weighted transverse MRI, **c** = CISS sequence reconstructed in sagittal plane, **d** = CISS sequence reconstructed in dorsal plane. In case 8, intra-dural spinal cord compression appears to be associated with an expansion of the dorsal arachnoid space, consistent with spinal arachnoid diverticula. CISS imaging highlights this CSF accumulation without apparent attachment of the dura to the pia (dashed line). In case 14, in a transverse plane (lower row image b) the spinal cord appears to develop a ‘stellate’ appearance with multiple hypo-intense bands that cross the arachnoid space (highlighted with short arrows on image b). At the corresponding level on CISS imaging (lower row, images c and d, dashed line) the hypo-intense bands appear lateral to the compressed spinal cord (level marked with dashed line) with an appearance reminiscent of the original description of ‘constrictive myelopathy’ [4]

**Fig. 3:**
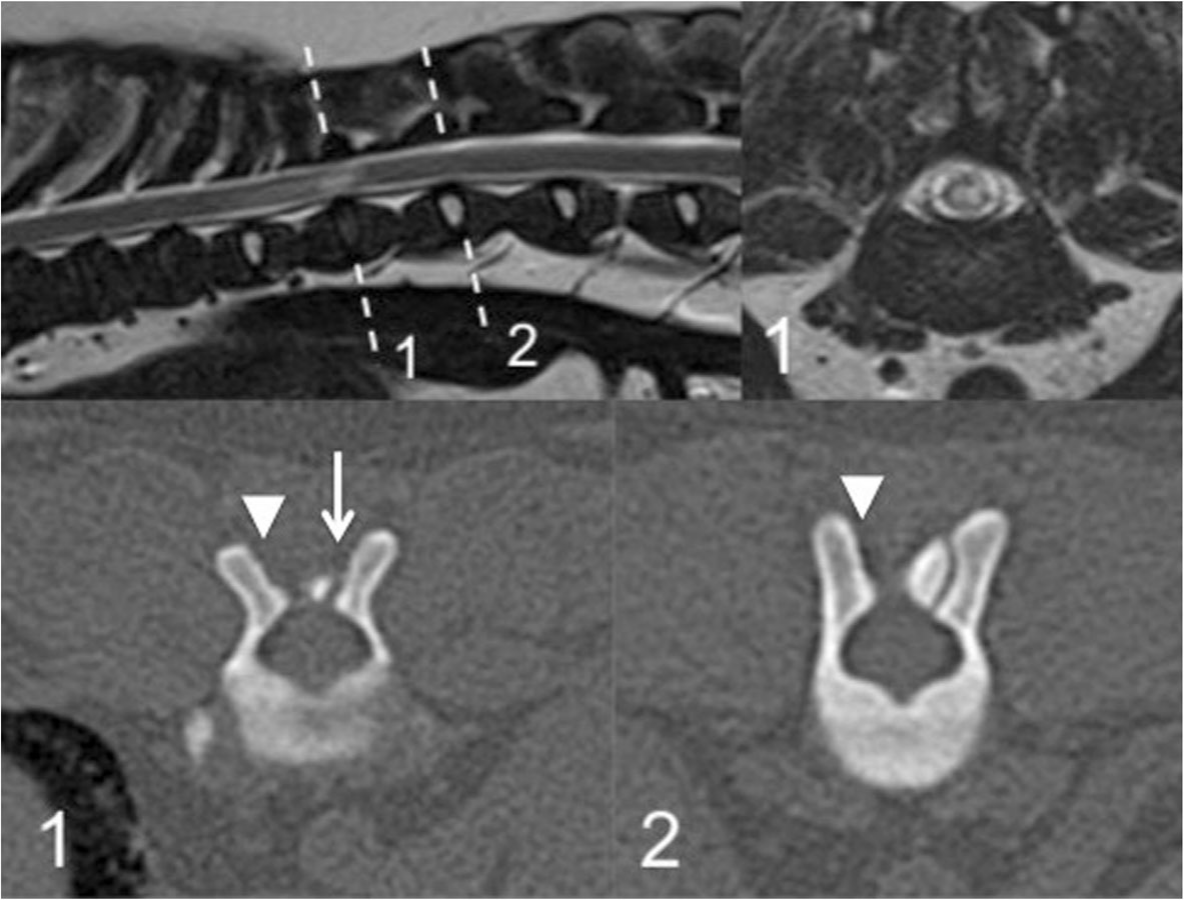
Magnetic resonance imaging and computed tomography of the thoracolumbar spine of case no. 13, which does not clearly display spinal cord compression. Top row left to right; sagittal T2-weighted image of the thoracolumbar spine, transverse T2-weighted image at the level of the spinal cord lesion at T11–12 (corresponding to dashed line labelled 1). Bottom row left to right; transverse computed tomography slice at affected level T11–12 (corresponding to dashed line labelled 1) displaying concurrent bilateral caudal articular process dysplasia (left-sided hypoplasia, arrowed, right-sided aplasia, arrow-head), transverse computed tomography slice at unaffected level T12–13 (corresponding to dashed line labelled 2) displaying unilateral (right-sided; arrow-head) caudal articular process aplasia

Imaging findings are summarised in Table 1. On MRI, a focal increase in T2-weighted signal was present within the spinal cord at the level of the other abnormalities described below. Widespread intervertebral disc degeneration was also present in all cases, but extra-dural spinal cord compression associated with intervertebral disc protrusion was only present in 10/18 (56%) of cases. In some of these cases the severity of extra-dural spinal cord compression was subjectively considered to be mild despite marked intra-medullary signal changes (Fig. 1). 7/18 cases displayed intra-dural spinal cord compression; in 4/18 cases (22%) this was associated with a significant expansion of the dorsal arachnoid space filled with CSF, suggestive of SAD (Fig. 2). In 3/18 cases (17%), MRI changes previously associated with pia-arachnoid fibrosis [14] were seen and this was recorded as the radiologic diagnosis (Fig. 2). In these cases, the spinal cord appeared to develop a ‘stellate’ appearance in a transverse plane with multiple hypo-intense bands crossing the arachnoid space (Fig. 2). At the corresponding level on CISS imaging (used to accentuate small structures surrounded by CSF) reconstructed in a dorsal plane, these hypo-intense bands appeared to obstruct the arachnoid space lateral to the spinal cord (Fig. 2). In one dog (case 13), focal and symmetric well-demarcated intramedullary signal changes were evident without spinal cord compression; vertebral instability was recorded as the putative diagnosis (Fig. 3). 6/18 (33%) cases were diagnosed with syrinx or pre-syrinx cranial to the site of the primary spinal cord lesion.

**Table 1:** Table summarising clinical and radiologic findings in Pug dogs with myelopathy apparently associated with caudal articular process (CAP) dysplasia. *SAD = spinal arachnoid diverticulum. **Vertebra affected (left/right) where a = aplastic, ‘h’ = hypoplastic and ‘n’ = normal

Study No.	Age (months)	Sex	Body weight (Kg)	Function score (0–15)	Duration of clinical signs (months)	Incontinence	Focal intra-medullary T2-weighted signal increase	Disc degeneration	Extra-dural compression Disc protrusion	Intra-dural compression: SAD*	Intra-dural compression: Pia-arachnoid fibrosis	Syrinx or pre-syrinx	Level of MRI Lesions	Level(s) of CAP dysplasia**
1	110	FN	8.20	8	5	Both	Yes	Yes	Yes	No	No	Yes	T12–13	T11a/a, T12a/a, T13 h/h
2	99	M	11.15	10	12	Urinary	Yes	Yes	No	No	Yes	No	T12–13	T10 h/a, T11a/a, T12 h/a
3	104	FN	7.95	11	1	None	Yes	Yes	Yes	No	No	No	T11–12	T11a/a
4	126	M	9.50	9	3	None	Yes	Yes	Yes	No	No	No	T11–12	T10a/a, T11a/a
5	144	FN	7.80	10	24	Faecal	Yes	Yes	Yes	No	No	No	T11–12	T11a/a, T12a/n
6	103	F	11.00	10	8	Both	Yes	Yes	Yes	No	No	No	T11–12	T10a/a, T11a/a
7	108	MN	10.40	11	16	Faecal	Yes	Yes	No	No	Yes	No	T10–11	T10a/a, T11 h/a, T12 h/n
8	102	FN	6.85	10	4	Faecal	Yes	Yes	No	Yes	No	Yes	T12–13	T10a/a, T11a/a, T12a/a
9	109	F	8.88	12	3	None	Yes	Yes	No	Yes	No	Yes	T11–12	T11a/a, T12n/h, T13n/h
10	112	M	10.05	9	30	Both	Yes	Yes	No	Yes	No	Yes	T10–11	T10a/a, T11 h/h
11	125	FN	7.70	10	24	None	Yes	Yes	No	Yes	No	No	T10–11	T10a/a
12	132	M	8.50	10	1	None	Yes	Yes	Yes	No	No	No	T11–12	T10 h/a, T11a/a, T12a/a
13	64	M	14.10	11	1	None	Yes	Yes	No	No	No	No	T11–12	T10a/a, T11 h/a, T12n/a
14	107	MN	9.50	9	15	Both	Yes	Yes	No	No	Yes	Yes	T10–11	T10a/a, T11n/h
15	83	FN	6.59	8	4	None	Yes	Yes	Yes	No	No	No	T12–13	T10a/a, T11 h/h, T12a/a
16	108	MN	7.90	11	5	None	Yes	Yes	Yes	No	No	Yes	T12–13	T10a/a, T11a/a, T12a/a, T13 h/n
17	111	M	9.30	11	2	None	Yes	Yes	Yes	No	No	No	T13-L1	T10a/a, T11a/a, T12a/a, T13a/h
18	60	M	7.20	10	6	Faecal	Yes	Yes	Yes	No	No	No	T11–12	T10a/a, T11a/a, T12a/a

**Fig. 4:**
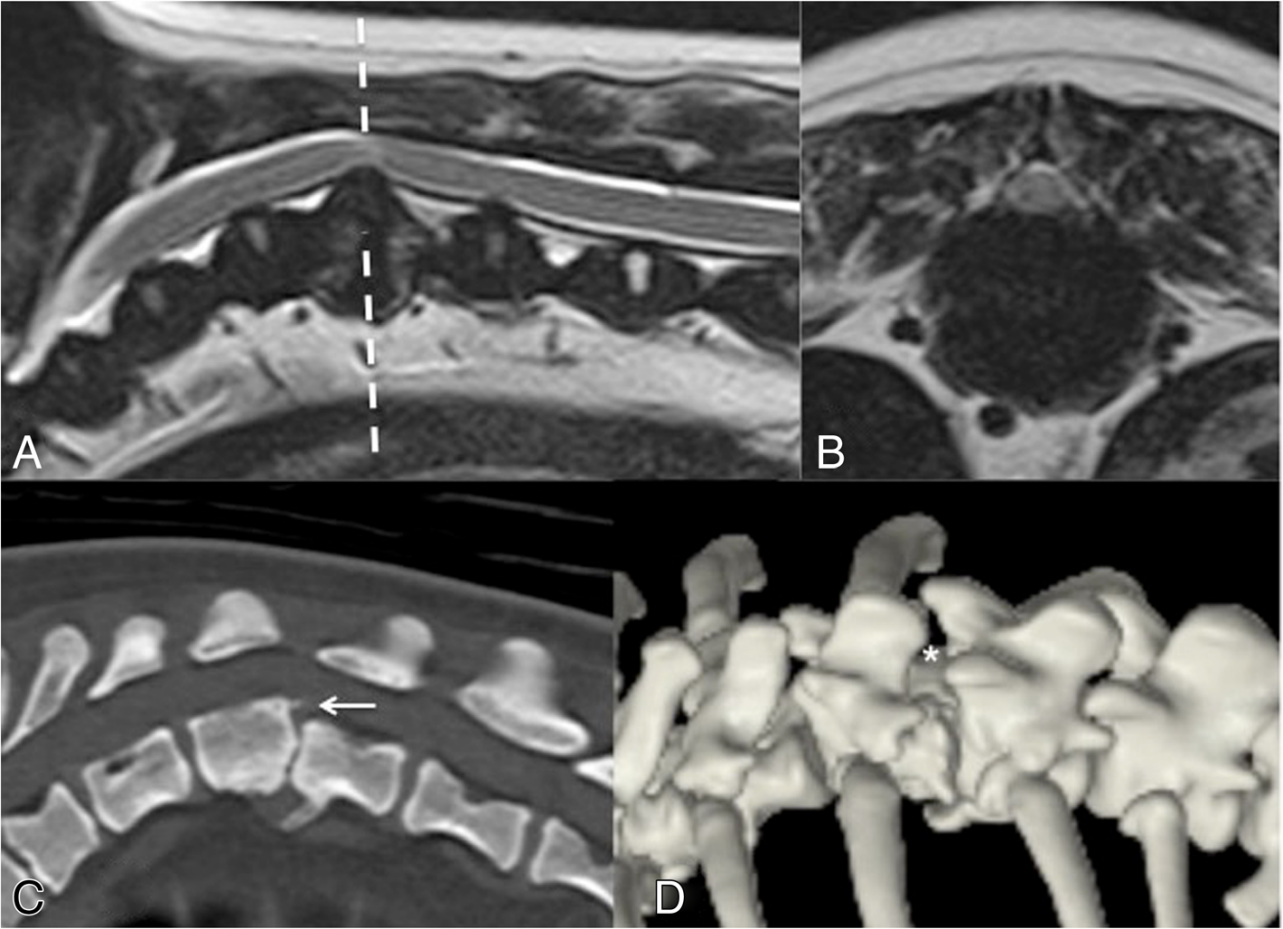
Magnetic resonance imaging and computed tomography of the thoracolumbar spine of case no. 18. **a** = T2-weighted sagittal MRI, **b** = T2-weighted transverse MRI at affected level T11–12 (corresponding to dashed line from image a), **c** = sagittal reconstruction of CT centred on T11–12, **d** = Three-dimensional reconstruction of CT viewed from a dorsolateral aspect. There is extra-dural spinal cord compression due to T11–12 intervertebral disc protrusion (images a and b). The vertebral column adopts a kyphotic appearance with marked narrowing of the T11–12 intervertebral disc space, with apposing vertebral end-plate sclerosis, ventral spondylosis deformans and slight retrolisthesis (image c; arrowed). There is bilateral aplasia of the caudal articular processes of T11 with an increase in the distance between the dorsal laminae of T11 and T12 (image d; asterisk)

On CT, bilateral CAP aplasia was present at the level of the MRI abnormalities described in 17 cases, with one case (case 13, Fig. 3) where one CAP was aplastic and the other hypoplastic. The level of bilateral CAP aplasia most commonly associated with MRI abnormalities was T11 (MRI abnormalities therefore evident at the T11–12 intervertebral disc space), in 8/18 (44%) of cases. Other levels associated with MRI abnormalities included T10 (4/18), T12 (5/18), and T13 (1/18). CAP dysplasia (unilateral or bilateral) was also present at a minimum of one other level in 16/18 (89%) of cases without concurrent MRI abnormalities (Table 1). CAP aplasia was common; of 87 individual dysplastic CAP examined, 18/87 (20.7%) were hypoplastic and 69/87 (79.3%) were aplastic. In two cases, only one thoracic vertebra displayed CAP dysplasia, which was bilateral aplasia, occurring at the affected level on MRI. One dog with bilateral CAP aplasia at T11 had a markedly kyphotic posture with marked reduction of the T11–12 intervertebral disc space, apposing vertebral end-plate sclerosis, ventral spondylosis deformans and slight retrolisthesis (Fig. 4).

There was no association between gender, neuter status, any form of incontinence and a diagnosis of intra-dural vs extra-dural compression groups (Table 2). Moreover, there was no difference in the body weight and clinical severity score at presentation (Table 3). There was weak evidence that compared to extra-dural compression, Pug dogs with an intra-dural compression had a longer duration of clinical signs at presentation to the clinic; but this did not reach statistical significance (mean duration 14.9 versus 5.9 months, *P* = 0.056).

**Table 2:** Association between categorical variables and the diagnosis of intra-dural or extra-dural spinal cord compression in Pug dogs with thoracolumbar myelopathy and concurrent caudal articular process dysplasia

Variable	Category	*N*	Intra-dural compression (*N*, row %)	Extra-dural compression (*N*, row %)	*P*-value
Sex	Male	9	4 (44.4)	5 (55.6)	*P* = 1.0
	Female	8	3 (37.5)	5 (62.5)	
Neuter status	Entire	6	2 (33.3)	4 (66.7)	*P* = 1.0
	Neutered	11	5 (45.5)	6 (54.6)	
Faecal incontinence	Yes	8	4 (50.0)	4 (50.0)	*P* = 0.64
	No	9	3 (33.3)	6 (66.7)	
Urinary incontinence	Yes	5	3 (60.0)	2 (40.0)	*P* = 0.59
	No	12	4 (33.3)	8 (66.7)	
Incontinence (faecal or urinary)	Yes	9	5 (55.6)	4 (44.4)	*P* = 0.34
	No	8	2 (25.0)	6 (75.0)

**Table 3:** Association between continuous variables and the diagnosis of intra-dural or extra-dural spinal cord compression in Pug dogs with thoracolumbar myelopathy and concurrent caudal articular process dysplasia

Variable	Intra-dural compression (median, range) *n* = 7	Extra-dural compression (median, range) *n* = 10	*P*-value
Weight (kg)	9.2 (6.9–11.2)	8.4 (6.5–11.0)	0.26
Duration of clinical signs (months)	14.9 (3.0–30.0)	5.9 (1–24)	0.056
Olby severity score	10.1 (9–12)	9.8 (8–11)	0.72

## Discussion

‘Lateral narrowing of the spinal cord through T11-L1’ was the only described MRI abnormality in a small number of Pug dogs with ‘constrictive myelopathy’, a condition previously attributed to concurrent CAP dysplasia [4]. This is the first study to describe the specific MRI findings in a group of Pug dogs with thoracolumbar myelopathy and concurrent CAP dysplasia. Our additional aims were determine the distribution and classification of dysplastic CAP and to examine relationships between clinical parameters and the presence of extra-dural vs intra-dural spinal cord compression. Four different putative diagnoses were made on the basis of MRI findings; intervertebral disc protrusion, SAD, pia-arachnoid fibrosis and vertebral instability. Although extra-dural spinal cord compression attributed to intervertebral disc disease was the most common putative diagnosis (56%), intra-dural compression was common (39%), as was faecal and urinary incontinence (50%). Pug dogs in this study were presented when slightly older, 9 years of age, than those from previously reported data concerning the general canine population affected by intervertebral disc disease [11, 15] and SAD [3].

A morphologic analysis of the canine spine has suggested there is a high prevalence of articular process dysplasia in neurologically normal small breed dogs [16] and CAP dysplasia appears to be very common in neurologically normal Pug dogs [9]. In this study, MRI abnormalities all occurred at an inter-vertebral level with bilaterally abnormal facet joints due to bilateral CAP aplasia in 17 cases and aplasia/hypoplasia in 1 case. In two dogs with just one level of aplastic CAP, MRI abnormalities occurred at that level. However, CAP dysplasia was also commonly evident at other levels in the T10–13 region. Our findings are in agreement of those of Rhodin et al., 2018, who recently described CAP dysplasia to be the single most common congenital vertebral malformation adjacent to focal spinal cord pathology in Pug dogs [17]. The authors also identified that CAP dysplasia was a common finding in Pug dogs regardless of neurologic deficits. Neurologically normal Pug dogs were previously found to display a greater tendency for CAP aplasia (as opposed to hypoplasia) in the post-diaphragmatic region of the thoracic spine [9] and our results support the notion that this may increase the likelihood of CAP dysplasia being clinically relevant in this breed.

It has previously been speculated that CAP dysplasia would result in instability of the vertebral column [4]. There may be other factors that contribute to potential instability at the affected level, such as prior trauma [4], an increased distance from the costovertebral articulations that provide stability to the mid-thoracic spine in axial rotation and lateral bending [18, 19], or other biomechanical considerations. Trauma was not recorded in this history of any Pug dogs in this study. Further biomechanical studies would be required to investigate whether CAP dysplasia is associated with vertebral instability and whether Pug dogs are likely to be at greater risk due to the nature and spatial distribution of their malformations. CAP dysplasia may be an unrelated epiphenomenon in Pug dogs with thoracolumbar myelopathy.

Intervertebral disc protrusion was the most common imaging diagnosis made in this study. In some Pug dogs, extra-dural spinal cord compression appeared subjectively mild considering the severity of the intra-medullary spinal cord changes. As the changes are focal, symmetric and relatively well-demarcated, such signal changes are suspected to represent oedema, inflammation, ischaemia, gliosis, and myelomalacia as has previously been suspected in humans and dogs with chronic cervical myelopathy [20, 21]. In one Pug dog, an increase in T2-weighted signal within the spinal cord was not associated with extra or intra-dural spinal cord compression. We theorised this lesion may be associated with vertebral instability or dynamic vertebral canal stenosis occurring with movement, as in other canine spinal diseases; for example, in dogs with canine cervical spondylomyelopathy, worsening of spinal cord compression can be demonstrated with the vertebral column in an extended or flexed position [22]. This theory has recently been further tested in Pug dogs using stress myelography [23]. Another dog in this study suffered intervertebral disc space collapse and vertebral retrolisthesis at T11–12 where CAP were aplastic, supporting CAP dysplasia could be associated with vertebral instability in some dogs.

39% of Pug dogs in the current study demonstrated intra-dural spinal cord compression on MRI; 22% of all dogs were putatively diagnosed with SAD. In the largest retrospective study regarding SAD in dogs, Pug dogs were the most commonly affected breed and were diagnosed at a significantly older age (median 59 months) than in other breeds [3]; however, this study included cervical SAD and did not record whether some of the Pug dogs suffered concurrent CAP dysplasia. This may in part be a consequence of the aforementioned difficulty in assessing CAP on MRI studies alone. It could be speculated that our smaller population of Pug dogs, which presented at an older age (median 108 months), than those previously reported [3], has identified Pug dogs with SAD more likely to have an acquired aetiology. The term ‘constrictive myelopathy’ has been used to describe an unusual cause of thoracolumbar myelopathy in Pug dogs characterised by the presence of fibrous pia-arachnoid adhesions which occurred in apparent association with dysplastic CAP [4]. 17% of Pug dogs in this study were diagnosed with pia-arachnoid fibrosis. The definition of this radiologic diagnosis was based on recent imaging and subsequent surgical descriptions, where histopathologic examination of adhesions revealed dense fibrous tissue and haemorrhage [14]. The authors described obliteration of the subarachnoid space, with neighboring regions of dilatation causing distortion of the cord. This was a feature of the three cases in our study. A multi-lobulated SAD would represent an important differential diagnosis, however these are suspected to represent a congenital variant and are only reported to occur in the cervical spine in commonly affected breeds [3, 24]. Nonetheless, it may be difficult to differentiate multilobulated SAD from pia-arachnoid fibrosis on the basis of MRI. The aetiologies, similarities and differences of acquired SAD and pia-arachnoid fibrosis will remain contentious as detailed imaging and histopathological descriptions are lacking. In humans, acquired fibrous arachnoid adhesions can form secondary to intra-arachnoid haemorrhage [25] inflammation [26] and rarely as a familial disease [27]. Low-grade instability associated with CAP dysplasia [4] and haemorrhage [14] has also been implicated in dogs. Similarly, SAD are often identified in dogs with previous or concurrent spinal cord disorders [3]. We therefore suspect pia-arachnoid fibrosis represents an acquired phenomenon that may share an inflammatory or traumatic aetiopathogenesis. Further study is required to ascertain whether Pug dogs have a tendency for familial arachnoid fibrosis.

Faecal and urinary incontinence was surprisingly common in this study, affecting 50% of the dogs. Incontinence has previously been reported in dogs with chronic upper motor neuron spinal cord lesions involving the dorsal aspects of the spinal cord [25], and particularly with spinal arachnoid diverticulae [28–30]. The dorsal location of arachnoid diverticulae in dogs may be important because of the potential disruption of the sensory pathways of defecation [13]. In one study that included five dogs with faecal incontinence undergoing spinal surgery, four were found to have arachnoid diverticulae [31]. However, several Pug dogs in this study were incontinent associated with intervertebral disc protrusion and there was no significant difference between groups. In humans, faecal incontinence can also be associated with upper motor neuron spinal cord lesions; anorectal sensory thresholds are increased in affected individuals compared with thresholds in unaffected controls, suggesting disruption of the afferent sensory pathways has occurred [30, 32].

The presence of CAP dysplasia has implications for surgical planning and technique. We advocate the use of both MRI and CT in affected Pug dogs to assess for the presence of bilateral CAP dysplasia at the level of spinal cord lesions. Stabilisation of the vertebral column may be appropriate in combination with techniques to alleviate extra or intra-dural spinal cord compression. A poor long-term outcome has been reported in Pug dogs with arachnoid fibrosis and diverticulae treated surgically [4, 14, 33]. However, this may be related to the chronicity of the myelopathy and spinal cord atrophy, rather than the technique employed. Further long-term follow up studies in dogs treated surgically are needed to determine which is more likely.

The main limitation to our study is the lack of surgical or histologic confirmation of our interpretation of the MRI abnormalities described. For example, although other authors have confirmed the MRI changes we report as pia-arachnoid fibrosis in surgery [14], these changes might reflect another pathology such as spinal cord atrophy or multi-lobulated SAD. However, given the appearance of the MRI abnormalities described, we suspect these cases best fit the original description of ‘constrictive myelopathy’ [4].

## Conclusions

Pug dogs with thoracolumbar myelopathy in this study were all middle-aged to older animals with chronic clinical signs often presenting with faecal or urinary incontinence. Pug dogs were often diagnosed with extra-dural spinal cord compression associated with intervertebral disc protrusion, however intra-dural spinal cord compression associated with SAD and pia-arachnoid fibrosis (‘constrictive myelopathy’) was also common. There was no association between clinical parameters and extra-dural vs intra-dural spinal cord compression. Bilateral CAP dysplasia (aplasia in all but one case) was always present at the level of MRI abnormalities, although also occurred at multiple other levels in the T10–13 region. CAP dysplasia occurred at multiple levels in the T10–13 region with T11 most often associated with MRI abnormalities. A causal relationship between CAP dysplasia and myelopathy is speculated but cannot be confirmed by this study.

## Abbreviations

CAP: Caudal articular process
CISS: Constructive Interference in Steady State
CSF: Cerebrospinal fluid
CT: Computed tomography
DICOM: Digital Imaging and Communications in Medicine
MRI: Magnetic Resonance Imaging
SAD: Spinal Arachnoid Diverticulum
SOD: Super-Oxide Dismutase
